# Predictors for Improvement of Global Functioning in Patients with Axial Spondyloarthritis

**DOI:** 10.3390/jcm14134474

**Published:** 2025-06-24

**Authors:** Kwi Young Kang, Brittany L. Adler, Tae Hwan Chung

**Affiliations:** 1 Department of Physical Medicine and Rehabilitation, Johns Hopkins University, Baltimore, MD 21224, USA; kykang@catholic.ac.kr; 2 Department of Internal Medicine, Incheon St. Mary’s Hospital, College of Medicine, The Catholic University of Korea, Seoul 06591, Republic of Korea; 3 Department of Medicine, Rheumatology Division, Johns Hopkins University, Baltimore, MD 21224, USA; brit.adler@jhmi.edu

**Keywords:** axial spondyloarthritis, ASAS health index, global functioning, BASFI, BASDAI

## Abstract

**Objectives**: We aimed to investigate the factors associated with changes in the global functioning of patients with axial spondyloarthritis (axSpA) and to identify predictors of improvement. **Methods**: One-hundred-and-eighty-five patients enrolled in the Incheon Saint Mary’s axSpA prospective observational cohort were evaluated. Global functioning was assessed at baseline and at 1-year follow-up using the ASAS health index (HI). Improvement was defined as a reduction in the ASAS HI of ≥3. Physical function was assessed using the Bath Ankylosing Spondylitis Functional Index (BASFI). Disease activity measures included Bath Ankylosing Spondylitis Disease Activity Index (BASDAI), Ankylosing Spondylitis Disease Activity Score (ASDAS), and C-reactive protein (CRP) levels. Predictors of improved global functioning were identified by logistic regression analysis. **Results**: Nineteen patients (10%) showed improved global functioning at 1-year follow-up versus baseline. Univariate linear regression analysis identified body mass index at baseline, the use of TNF inhibitors, a change in the BASFI and BASDAI, and changes in CRP levels as being associated with changes in the ASAS HI. Multivariate analysis revealed that changes in the BASFI and BASDAI were associated independently with a change in the ASAS HI. Univariate logistic regression analysis revealed that a decrease in the BASFI, BASDAI, ASDAS, and CRP levels predicted improved global functioning. Multivariate analysis identified a decrease in the BASFI and BASDAI as a significant predictor of improved global functioning (odds ratio (95% CI) = 1.465 (1.006–2.135) and 1.414 (1.044–1.914), respectively). **Conclusions**: Changes in physical function and disease activity were associated independently with changes in global functioning assessed by the ASAS HI in axSpA. A decrease in the BASFI and BASDAI was a significant predictor of improvement in the ASAS HI.

## 1. Introduction

Axial spondyloarthritis (AxSpA) is a chronic inflammatory disease that primarily affects the axial skeleton. AxSpA can be divided into radiographic axSpA (r-axSpA), also known as ankylosing spondylitis (AS), and non-radiographic axial spondylarthritis (nr-axSpA). Patients with r-axSpA have already developed structural damage in the sacroiliac joints or spine which is visible on radiographs, while patients with nr-axSpA do not have such structural damage. Patients with axSpA present with chronic back pain and stiffness, predominantly in the pelvis and lower back area, but also in the peripheral joints, which can cause arthritis and enthesitis [1]. These various axial and peripheral joint symptoms limit daily activities and social participation [2].

The Assessment of Spondyloarthritis International Society (ASAS)/European Alliance of Associations for Rheumatology (EULAR) recommends that the primary goal of treating patients with axSpA is to maximize health-related quality of life by controlling symptoms and inflammation, preventing progressive structural damage, and preserving/normalizing function and social participation [3]. However, the overall impact of disease activity on impairment, limitation, and restriction in activities or social participation is not adequately assessed by SpA-specific instruments [4]. Therefore, it is important to use disease-specific health-related quality of life measures in order to assess whether treatment for axSpA is meaningfully addressing functional impairment and social participation in real-world clinical practice.

The ASAS health index (HI) is a patient-reported outcome measure based on the International Classification of Functioning, Disability, and Health, commonly known as the ICF. The ICF is a framework established by the World Health Organization (WHO) for measuring health and disability at both the individual and population levels. According to the ICF, disability is a decrement in any functional domain, including cognition, mobility, self-care, getting along, life activities, and participation. The idea behind the development of the ASAS HI is to cover all possible limitations in global functioning experienced by patients with spondyloarthritis (SpA) based on the ICF [5,6]. The ICF model provides holistic and biopsychosocial perspectives of disability, which were not present in previous SpA-specific instruments. The ASAS HI includes 17 dichotomous items that represent different categories, including pain, emotional function, sleep, sexual function, mobility, independence, social life, and working life.

The ASAS HI is currently recommended as a core outcome instrument for assessing overall function and health in patients with axial spondyloarthritis (axSpA) [7]. Also, the ASAS HI was chosen as the primary outcome measure for the first treat-to-target (T2T) strategy trial in axSpA, referred to as the Tight Control in Spondyloarthritis (TICOSPA) study [8]. Since the ASAS HI was first introduced, it has been emerging as an important tool for axSpA research [9].

Some of the advantages of the ASAS HI are that it is fast, simple, and accessible, allowing it to be used in clinical practice [10]. However, its use for axSpA research remains limited. Most previous research studies that used the ASAS HI were cross-sectional in design [5,6,9,11,12]. Others analyzed the sensitivity of the ASAS HI to functional changes in patients who initiated therapy or changed from their original therapy [5,9,11,13], and, so far, a few clinical trials have used it to assess the effects of treatment on function and health [14,15]. To date, no study has investigated the factors that predict improvement in ASAS HI scores for patients with axSpA using longitudinal data in clinical practice.

In this longitudinal study, we aimed to identify the factors that drive changes in the ASAS HI among patients with axSpA, with a primary goal of predicting significant improvements in ASAS HI scores.

## 2. Materials and Methods

### 2.1. Study Patients

The Incheon Saint Mary’s AXial SPondyloArthritis study (ISAXSPA) is a prospective observational cohort study in which patients are followed according to a fixed protocol [16]. This study used ISAXSPA data collected from 2014. Patients recruited into this cohort were required to fulfil the ASAS axSpA criteria [17], including patients with established AS [18]. Patients were evaluated annually according to a standardized protocol that included clinical, laboratory, and imaging variables. Patient questionnaire data (including information regarding classification, comorbidity, disease activity, and functional assessments) were evaluated at baseline and annually.

The present study analyzed 185 consecutive patients from 2014 who had at least two sets of ASAS HI data, physical function questionnaire data, and data regarding disease activity. All patients were aged ≥ 18 years. The study was performed in accordance with the Declaration of Helsinki. Written informed consent was obtained from all study participants prior to inclusion in the observational cohort, and the study protocol was approved by the local ethics committee (study number OC16OISI0138). This study conforms to all STROBE guidelines and reports the required information accordingly (see Supplementary Checklist Table S1).

### 2.2. Clinical Variables

Sex, age, time post-symptom onset, and the presence of HLA B27 were recorded at the time of enrollment. The following clinical data were collected annually at the same visit as the ASAS HI assessment was completed: body mass index, current smoking status, number of alcoholic drinks per week, disease activity measures, physical function assessment, and current medications. The use of nonsteroidal anti-inflammatory drugs (NSAIDs), sulfasalazine, and tumor necrosis factor (TNF) inhibitors was recorded as a dichotomous variable (yes/no) during every year of follow-up, with patients who had taken agents for a period of 6 months or longer being considered as “sustained users”.

Measures of disease activity were collected using the Bath Ankylosing Spondylitis Disease Activity Index (BASDAI) [19] and the Ankylosing Spondylitis Disease Activity Score (ASDAS) [20]. Erythrocyte sedimentation rate (ESR) and C-reactive protein (CRP) levels were also measured. Physical function was measured using the Bath AS functional index (BASFI) [21].

Radiographic sacroiliitis on anterior–posterior views of the pelvis at the time of first ASAS HI assessment was assessed according to the modified New York criteria [18]. The number of syndesmophytes on lateral views of the cervical and lumbar spine was assessed using the modified Stoke Ankylosing Spondylitis Spinal Score [22]. Radiographs were scored by a single trained expert who was blinded to the patients’ characteristics.

### 2.3. Assessment of Global Functioning

Global functioning was assessed using the ASAS HI. In ISAXSPA, the validated Korean version of the ASAS HI is used [23]. This instrument comprises 17 items, expressed in the first person and in the present tense, with the following dichotomous response options: “I agree” or “I do not agree.” Each positive answer is scored 1 and each negative answer is scored 0. The result is the sum of all individual items [6]. The sum score of the ASAS HI ranges from 0 (good functioning) to 17 (poor functioning). Based on thresholds, global functioning is categorized as good (ASAS HI ≤ 5), moderate (ASAS HI > 5 and <12), or poor (ASAS HI ≥ 12) [5]. In previous studies, the smallest detectable change was calculated as 3.0, which corresponds to the minimum change (beyond measurement error) that can be detected in an individual patient over time [5,9]. In the present study, improvement was defined as a decrease in the ASAS HI of ≥3, deterioration was defined as an increase of ≥3 or more, and maintenance was defined as a change of <3.

### 2.4. Statistical Analysis

Descriptive data are presented as absolute frequencies and percentages. Continuous variables are expressed as the mean ± standard deviation. A paired *t*-test was used to compare continuous variables at baseline and at 1-year follow-up. Correlations between changes in the ASAS HI and changes in disease variables were assessed by calculating the Pearson’s correlation coefficient. Linear regression models were used to examine the factors associated with a change in the ASAS HI at 1 year. All variables significant in univariate linear regression analyses (*p* < 0.1) were entered into multivariate analyses as explanatory variables. Parameter estimates (β) were calculated with 95% confidence intervals (CI). Univariate and multivariate logistic regression analyses were performed to identify the factors that predict improvement in the ASAS HI. Odds ratios (ORs) with 95% CIs were calculated. Variables identified by univariate logistic regression analysis (*p* < 0.1) were included in a multivariate logistic regression model (stepwise method).

All statistical tests were two tailed, and statistical significance was defined as *p* < 0.05. All statistical analyses were performed using SPSS (version 21.0; IBM Corp., Armonk, NY, USA).

## 3. Results

Table 1 shows the demographic and clinical characteristics of the 185 patients at baseline. Among them, the mean age was 37 years, 84% were male, and 89% were HLA-B27-positive. The mean ASAS HI was 2.7. The average score for the BASFI, BASDAI, and ASDAS were 0.9, 2.3, and 1.7, respectively. Sacroiliitis on X-ray, which fulfilled the modified New York criteria for the classification of AS, was observed in 128 (69%) patients. The average modified Stoke Ankylosing Spondylitis (mSASSS) was 7.7, and the mean number of syndesmophytes was 3.1.

Table 2 presents the mean ASAS HI, BASFI, and disease activity measures at baseline and 1-year follow-up. There was a significant decrease in the ASAS HI, BASFI, BASDAI, and ASDAS scores at 1-year follow-up compared with baseline. CRP decreased at 1-year follow-up, but the change was not significant (*p*-value = 0.061).

**Figure 1:**
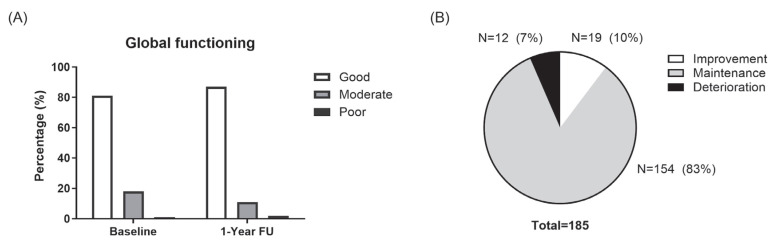
Changes in global function at 1 year. (**A**). Distribution of global functioning levels (good, moderate, and poor) at baseline and at the 1-year follow-up. (**B**). The proportion of patients with improved (≥3-point decrease), maintained (<3-point change), or worsened (≥3-point increase) ASAS HI scores over 1 year.

The overall distribution of global functioning severity in the cohort remained largely consistent between baseline and 1-year follow-up (Figure 1). The proportion of patients with good, moderate, or poor global function by ASAS HI score (as good (ASAS HI ≤ 5), moderate (ASAS HI > 5 and <12), or poor (ASAS HI ≥ 12)) was 151 (81%), 33 (18%), and 1 (1%) at baseline, and 161 (87%), 21 (11%), and 3 (2%) at 1-year follow-up, respectively (Figure 1A). Upon individual assessment of change, nineteen patients (10%) showed improved global functioning (decrease ≥ 3) at 1-year follow-up. Global functioning deteriorated in 12 patients (7%) (increase ≥ 3) and was maintained in 154 patients (83%) (Figure 1B).

Table 3 shows the correlation between changes in the ASAS HI and changes in the BASFI, BASDAI, ASDAS, and CRP. Changes in the ASAS HI correlated positively with changes in the BASFI, BASDAI, and CRP (r = <0.001, <0.001, and 0.047, respectively), but not the ASDAS.

Table 4 presents the results of linear univariate and multivariate regression analyses of changes in the ASAS HI. Univariate analysis identified body mass index at baseline, TNF inhibitor use, and changes in the BASFI, BASDAI, and CRP as being associated with changes in the ASAS HI. Changes in the ASDAS showed an equivocal association. Changes in the BASDAI correlated with changes in the ASDAS and changes in CRP levels (r = 0.590 and r = 0.306, respectively). Therefore, among disease activity measures, only changes in the BASDAI were included in multivariate analysis to avoid multicollinearity. Multivariate analysis identified changes in the BASFI and BASDAI as being associated independently with changes in the ASAS HI (β (95% CI) = 0.330 (0.062–0.598) and 0.326 (0.134–0.519), *p*-value = 0.016 and 0.001, respectively).

Table 5 shows the results of the logistic regression analysis of factors that predict improved global functioning. Univariate logistic regression analysis identified decreases in the BASFI, BASDAI, ASDAS, and CRP level as predictors of improved global function. The TNF inhibitor showed equivocal results. The results of the multivariate analysis showed that a one-point reduction in functional impairment (BASFI) significantly increased the odds of improved global functioning by 1.465 times (odds ratio (95% CI) = 1.465 (1.006–2.135)), as assessed by the ASAS HI. Likewise, a one-point decrease in disease activity (BASDAI) was associated with a 1.414-fold increase in the likelihood of improved global functioning ((odds ratio (95% CI) = 1.414 (1.044–1.914)).

We think that these findings underscore the importance of targeting both functional ability and disease activity in the management of patients with axial spondyloarthritis. Our results align with previous cross-sectional studies showing that BASDAI and BASFI scores are associated with better patient-reported outcomes, and they highlight the clinical value of comprehensive treatment strategies that address both domains.

## 4. Discussion

The present study used data from a prospective observational cohort study to analyze the factors that predict improvement in global functioning among patients with axSpA. About 10% of axSpA patients showed improved global functioning, as assessed by the ASAS HI. This relatively low responder rate may reflect the real-world challenges of achieving substantial improvements in global functioning in a cohort with low overall disease activity. It also highlights the need for sensitive prognostic tools and emphasizes that even small improvements in physical function and disease activity can meaningfully impact patient outcomes. Nevertheless, the limited number of responders may affect the robustness and generalizability of the predictive model, which we acknowledge as a limitation. Changes in global functioning were significantly associated with changes in physical function and disease activity. Reductions in the BASFI and disease activity measures were independent predictors.

The ASAS HI is a recently introduced health instrument that aims to evaluate the global functioning of patients with SpA based on the ICF model of disability. The term “functioning”, according to the ICF, encompasses all body functions, activities, and participation [4]. Prior to the development of the ASAS HI, SpA-specific instruments lacked an overall picture of disability that included impairments, limitations, and restrictions in activities or social participation [24]. The ASAS HI was developed with the specific aim of reflecting the common and holistic aspects of functioning that are important to patients with axSpA [25]. Higher ASAS HI scores reflect major impairment, limitation, and restriction [6,24]. The 17 items contained within the ASAS HI include pain, maintaining a body position, mobility, motivation, emotional function, sleep, sexual function, self-care, and community life. The ASAS HI effectively gathers all of these measurements, making it useful for routine clinical practice because it captures not only aspects related to the individual, but also to their social environment [4]. The ASAS HI is also used in clinical trials as a tool to assess the effects of biologic agents on the function and health of patients with axSpA. In both r-axSpA and nr-axSpA, patients treated with ixekizumab show significant improvement in the ASAS HI from baseline (an improvement of ≥ 3 from baseline, the same as reported in the current study) [14,15].

Cross-sectional studies have reported strong correlations between the BASFI and ASAS HI (*r* = 0.65–0.80) [5,11,12,23,26,27]. Patients with limited physical function had poor global functioning, as measured by the ASAS HI [5,11]. Our results at baseline also showed a positive correlation between the BASFI and ASAS HI (r = 0.602 and *p* < 0.001). In this longitudinal study, changes in the ASAS HI correlated significantly with changes in the BASFI, and multivariate regression analysis showed that a decrease in the BASFI was a significant predictor of improved global functioning, even after adjusting for changes in disease activity. These results indicate that improvements in physical function have a significant impact on subjective improvements in the quality of life of patients with axSpA.

Our data also showed that a reduction in the BASDAI was an independent predictor of improved global functioning. Multivariate analysis showed that changes in the ASAS HI correlated with changes in the BASDAI and were associated independently with changes in the BASDAI. This is consistent with the results from earlier cross-sectional studies showing that the ASAS HI correlates positively with disease activity measures such as the BASDAI (r = 0.58–0.77) and ASDAS (r = 0.50–0.70) [5,11,12,23,26,27].

Interestingly, our linear regression analysis results showed that changes in the BASDAI were associated with changes in the ASAS HI, but not in the ASDAS. This suggests that patient-reported disease activity measures have a significant effect on global functioning, and that this effect is potentially greater than that of objective parameters, given that the ASAS HI is a self-reported outcome, whereas the ASDAS is a composite measure of patient-reported outcomes and inflammatory biomarkers, which are objective parameters. It is still possible to pinpoint inflammatory biomarkers that better align with self-reported outcome measures like the ASAS HI. Recent breakthroughs in understanding the immune mechanisms behind axSpA are promising. For instance, the activation of IL-23- and IL-23-induced inflammatory immune cells is a central pathological mechanism in axSpA, with IL-17 and TNF-α serving as key effector cytokines [28]. Recent studies have also shown that macrophage migration inhibitory factor (MIF) and its receptor CD74 are extensively involved in the pathogenesis of SpA, contributing to inflammation in the spine, joints, eyes, skin, and gastrointestinal tract [29]. Persistent inflammation in axSpA can lead to spinal ankylosis [1], which significantly reduces quality of life and imposes a considerable socioeconomic burden, both directly and indirectly [30]. Subjective assessments of disease activity, such as the BASDAI, a patient-reported outcome, do not correlate markedly with objective parameters such as MRI inflammation scores [31]. This suggests that the BASDAI reflects more subjective opinions of disease activity than the ASDAS. Although the ASDAS has better psychometric properties than the BASDAI and is the preferred measure [32], the BASDAI appears to have an advantage in predicting functional outcomes, likely because it better reflects patients’ perspectives on disease activity.

It is also noteworthy that structural damage in the spinal structure had no significant effects on global function in the current study. Other studies report inconsistent results regarding the association between spinal structural damage and the ASAS HI [26,27]. One study reported a significant association between spinal structural damage and the ASAS HI [26], whereas a recent study of German patients reported that the ASAS HI was not influenced by structural damage [27]. Here, we found no association between structural damage and global functioning at baseline. Radiographic sacroiliitis showed no significant association with changes in the ASAS HI.

The study has several limitations. As mentioned in the methods section, this study analyzed data from an observational cohort, not a clinical trial. Therefore, there was no consistency regarding the time of ASAS HI assessment and the start date of treatment. This must be considered when interpreting the influence of treatment agents on improvements in global functioning. The effect of medications on changes in global function should be investigated using data from clinical trials or registries of patients starting new agents such as biologic agents. In particular, the treatment initiation dates of TNF inhibitors were not systematically recorded, so we could not distinguish between new and ongoing TNF inhibitor use. This limitation may have attenuated our ability to detect treatment effects on global functioning and should be addressed in future prospective studies. Additionally, depression is associated with impaired global functioning [27], but we had no baseline information about depressive symptoms. Therefore, we could not assess the effect of concomitant depression on changes in the ASAS HI. The absence of these variables is a notable limitation, and future studies incorporating mental health assessments are warranted. Lastly, some odds ratios in the multivariate analysis, such as for BASFI (OR 1.47; 95% CI 1.01–2.14), had relatively wide confidence intervals, suggesting potential variability and limited statistical power. These findings should be interpreted with caution and validated in larger cohorts.

## 5. Conclusions

Changes in both physical function and disease activity independently impact changes in global functioning, as captured by the ASAS HI in patients with axSpA. Among the disease activity measures examined (BASDAI, ASDAS, and CRP), only changes in the BASDAI were significant predictors of ASAS HI improvement. Furthermore, both reductions in BASFI and BASDAI were identified as significant independent predictors. These findings underscore the clinical value of adopting comprehensive treatment strategies that simultaneously target improvements in both physical function and disease activity to enhance the overall global functioning in patients with axSpA.icf.

## Figures and Tables

**Table 1:** Patient characteristics at baseline (N = 185).

Variable	Total (N = 185, N (%) or Mean ± SD)
Age, years	37.4 ± 10.8
Male	155 (84)
Body mass index, kg/m^2^	24.7 ± 3.9
Symptom duration, years	9.7 ± 7.9
HLA-B27-positive	164 (89)
Current smoking	77 (42)
Alcohol drinking, days of alcohol consumption per week	1.0 ± 1.1
ASAS HI (0–17)	2.7 ± 2.9
BASFI (0–10)	0.9 ± 1.3
BASDAI (0–10)	2.3 ± 1.9
ASDAS	1.7 ± 1.2
CRP, mg/L	4.6 ± 9.1
Radiographic sacroiliitis	128 (69)
mSASSS	7.7 ± 12.0
Number of syndesmophytes	3.1 ± 5.6
NSAIDs	173 (94)
Sulfasalazine	93 (50)
TNF inhibitors	37 (20)

ASAS HI, Assessment of Spondyloarthritis International Society health index; BASFI, Bath Ankylosing Spondylitis Functional Index; BASDAI, Bath Ankylosing Spondylitis Disease Activity Index; ASDAS, Ankylosing Spondylitis Disease Activity Score; CRP, C-reactive protein; mSASSS, modified Stoke Ankylosing Spondylitis Spinal Score; TNF, tumor necrosis factor.

**Table 2:** Comparison of outcome measures and ASAS HI scores at baseline and at 1-year follow-up in the patients studied.

Variable	Baseline	1 Year Follow-Up	*p*-Value
ASAS HI (0–7)	2.7 ± 2.9	2.4 ± 2.7	0.03
BASFI score (0–10)	0.9 ± 1.3	0.7 ± 1.1	<0.01
BASDAI score (0–10)	2.3 ± 1.9	1.9 ± 1.5	<0.01
ASDAS	1.7 ± 1.2	1.3 ± 0.9	<0.01
CRP, mg/L	4.6 ± 9.1	3.3 ± 6.0	0.06

ASAS HI, Assessment of Spondyloarthritis International Society health index; BASFI, Bath Ankylosing Spondylitis Functional Index; BASDAI, Bath Ankylosing Spondylitis Disease Activity Index; ASDAS, Ankylosing Spondylitis Disease Activity Score; CRP, C-reactive protein.

**Table 3:** Correlation between changes in the ASAS HI score and changes in disease variables.

	Changes of ASAS HI Score	
	r	*p*-Value
Changes in the BASFI score (0–10)	0.31	<0.01
Changes in the BASDAI score (0–10)	0.37	<0.01
Changes in the ASDAS	0.12	<0.01
Changes in CRP level, mg/L	0.15	0.047

ASAS HI, Assessment of Spondyloarthritis International Society health index; BASFI, Bath Ankylosing Spondylitis Functional Index; BASDAI, Bath Ankylosing Spondylitis Disease Activity Index; ASDAS, Ankylosing Spondylitis Disease Activity Score; CRP, C-reactive protein.

**Table 4:** Linear regression analysis of changes in ASAS health index scores in the analyzed patients.

Variables	Univariate Analysis		Multivariate Analysis #	
	β (95% CI)	*p*-Value	β (95% CI)	*p*-Value
Age, years	0.01 (−0.02–0.03)	0.70		
Male sex	0.45 (−0.33–1.25)	0.27		
Body mass index, kg/m^2^	0.09 (0.01–0.16)	0.02	0.06 (−0.00–0.13)	0.08
Current smoking	0.16 (−0.43–0.76)	0.60		
Alcohol drinking, days of alcohol consumption per week	−0.05 (−0.32–0.23)	0.75		
HLA-B27-positive	0.34 (−0.58–1.27)	0.47		
Radiographic sacroiliitis	−0.37 (−1.00–0.27)	0.26		
Number of syndesmophytes	−0.04 (−0.09–0.02)	0.17		
NSAIDs	0.21 (−0.72–1.13)	0.66		
Sulfasalazine	−0.29 (−0.87–0.30)	0.34		
TNF inhibitor	0.64 (0.00–1.28)	0.048	0.43 (−0.15–1.04)	0.14
Change in BASFI score	0.56 (0.32–0.81)	<0.01	0.33 (0.06–0.60)	0.02
Change in BASDAI score	0.47 (0.30–0.64)	<0.01	0.33 (0.13–0.52)	<0.01
Change in ASDAS	0.24 (−0.04–0.51)	0.10		
Change in CRP, mg/L	0.03 (0.00–0.06)	0.04		

NSAIDs, nonsteroidal anti-inflammatory drugs; TNF, tumor necrosis factor; BASFI, Bath Ankylosing Spondylitis Functional Index; BASDAI, Bath Ankylosing Spondylitis Disease Activity Index; ASDAS, Ankylosing Spondylitis Disease Activity Score; CRP, C-reactive protein. # adjusted for body mass index, TNF inhibitor use, changes in the BASFI score and changes in the BASDAI score.

**Table 5:** Logistic regression analysis of changes in global functioning as expressed by the ASAS HI score.

Variables	Univariate Analysis		Multivariate Analysis #	
	Odds Ratio (95% CI)	*p*-Value	Odds Ratio (95% CI)	*p*-Value
Age, years	1.01 (0.97–1.06)	0.65		
Male sex	1.73 (0.38–7.89)	0.48		
Body mass index, kg/m^2^	1.08 (0.96–1.21)	0.22		
Current smoking	1.64 (0.63–4.26)	0.31		
Alcohol drinking, days of alcohol consumption per week	1.05 (0.68–1.63)	0.82		
HLA-B27-positive	1.10 (0.24–5.13)	0.91		
Radiographic sacroiliitis	0.58 (0.22–1.52)	0.27		
Number of syndesmophytes	0.97 (0.87–1.08)	0.56		
NSAIDs	2.47 (0.31–19.49)	0.39		
Sulfasalazine	0.67 (0.25–1.80)	0.43		
TNF inhibitor	2.35 (0.90–6.15)	0.08		
Change in BASFI score	1.80 (1.28–2.51)	<0.01	1.47 (1.01–2.14)	0.047
Change in BASDAI score	1.64 (1.25–2.15)	<0.01	1.41 (1.04–1.91)	0.03
Change in ASDAS	1.87 (1.25–2.81)	<0.01		
Change in CRP, mg/L	1.05 (1.01–1.09)	0.02		

NSAIDs, nonsteroidal anti-inflammatory drugs; TNF, tumor necrosis factor; BASFI, Bath Ankylosing Spondylitis Functional Index; BASDAI, Bath Ankylosing Spondylitis Disease Activity Index; ASDAS, Ankylosing Spondylitis Disease Activity Score; CRP, C-reactive protein. # adjusted for TNF inhibitor use, changes in the BASFI score and changes in the BASDAI score.

## Data Availability

The data presented in this study are available on request from the corresponding author.

## Supplementary Materials

The following supporting information can be downloaded at: https://www.mdpi.com/article/10.3390/jcm14134474/s1. Table S1. STROBE Statement for cohort studies.

## References

[1] Sieper J., Poddubnyy D. (2017). Axial spondyloarthritis. Lancet.

[2] Dagfinrud H., Kjeken I., Mowinckel P., Hagen K.B., Kvien T.K. (2005). Impact of functional impairment in ankylosing spondylitis: Impairment, activity limitation, and participation restrictions. J. Rheumatol..

[3] Ramiro S., Nikiphorou E., Sepriano A., Ortolan A., Webers C., Baraliakos X., Landewé R.B.M., Van den Bosch F.E., Boteva B., Bremander A. (2023). ASAS-EULAR recommendations for the management of axial spondyloarthritis: 2022 update. Ann. Rheum. Dis..

[4] Alonso S., Morante I., Alperi M., Queiro R. (2022). The ASAS Health Index: A New Era for Health Impact Assessment in Spondyloarthritis. J. Rheumatol..

[5] Kiltz U., van der Heijde D., Boonen A., Akkoc N., Bautista-Molano W., Burgos-Vargas R., Wei J.C., Chiowchanwisawakit P., Dougados M., Duruoz M.T. (2018). Measurement properties of the ASAS Health Index: Results of a global study in patients with axial and peripheral spondyloarthritis. Ann. Rheum. Dis..

[6] Kiltz U., van der Heijde D., Boonen A., Cieza A., Stucki G., Khan M.A., Maksymowych W.P., Marzo-Ortega H., Reveille J., Stebbings S. (2015). Development of a health index in patients with ankylosing spondylitis (ASAS HI): Final result of a global initiative based on the ICF guided by ASAS. Ann. Rheum. Dis..

[7] Navarro-Compán V., Boel A., Boonen A., Mease P.J., Dougados M., Kiltz U., Landewé R.B.M., Baraliakos X., Bautista-Molano W., Chiowchanwisawakit P. (2022). Instrument selection for the ASAS core outcome set for axial spondyloarthritis. Ann. Rheum. Dis..

[8] Molto A., López-Medina C., Van den Bosch F.E., Boonen A., Webers C., Dernis E., van Gaalen F.A., Soubrier M., Claudepierre P., Baillet A. (2021). Efficacy of a tight-control and treat-to-target strategy in axial spondyloarthritis: Results of the open-label, pragmatic, cluster-randomised TICOSPA trial. Ann. Rheum. Dis..

[9] Regierer A.C., Weiß A., Kiltz U., Sieper J., Schwarze I., Bohl-Bühler M., Kellner H., Poddubnyy D., Zink A., Braun J. (2023). The Sensitivity to Change of the ASAS Health Index in an Observational Real-Life Cohort Study. J. Rheumatol..

[10] Queiro R., Alonso-Castro S., Alperi M. (2021). ASAS Health Index as an Addition to Routine Clinical Practice. J. Rheumatol..

[11] Rodrigues-Manica S., Cruz E., Ramiro S., Sousa S., Aguiar R., Sepriano A., Machado P., Branco J., Kiltz U., Pimentel-Santos F. (2020). The European Portuguese version of the ASAS Health Index for Patients with Spondyloarthritis: Measurement properties. Acta Reum. Port..

[12] Alonso-Castro S., Pardo E., Charca L., Pino M., Fernández S., Alperi M., Arboleya L., Queiro R. (2020). Performance of the ASAS Health Index for the Evaluation of Spondyloarthritis in Daily Practice. J. Rheumatol..

[13] Bautista-Molano W., Landewé R.B.M., Kiltz U., Valle-Oñate R., van der Heijde D. (2018). Validation and reliability of translation of the ASAS Health Index in a Colombian Spanish-speaking population with spondyloarthritis. Clin. Rheumatol..

[14] Walsh J.A., Magrey M.N., Baraliakos X., Inui K., Weng M.Y., Lubrano E., van der Heijde D., Boonen A., Gensler L.S., Strand V. (2022). Improvement of Functioning and Health with Ixekizumab in the Treatment of Active Nonradiographic Axial Spondyloarthritis in a 52-Week, Randomized, Controlled Trial. Arthritis Care Res..

[15] Kiltz U., Wei J.C., van der Heijde D., van den Bosch F., Walsh J.A., Boonen A., Gensler L.S., Hunter T., Carlier H., Dong Y. (2021). Ixekizumab Improves Functioning and Health in the Treatment of Radiographic Axial Spondyloarthritis: Week 52 Results from 2 Pivotal Studies. J. Rheumatol..

[16] Kang K.Y., Ju J.H., Park S.H., Hong Y.S. (2020). Longitudinal Association Between Trabecular Bone Loss and Disease Activity in Axial Spon-dyloarthritis: A 4-year Prospective Study. J. Rheumatol..

[17] Rudwaleit M., Landewé R., van der Heijde D., Listing J., Brandt J., Braun J., Burgos-Vargas R., Collantes-Estevez E., Davis J., Dijkmans B. (2009). The development of Assessment of SpondyloArthritis international Society classification criteria for axial spondyloarthritis (part I): Classification of paper patients by expert opinion including uncertainty appraisal. Ann. Rheum. Dis..

[18] van der Linden S., Valkenburg H.A., Cats A. (1984). Evaluation of diagnostic criteria for ankylosing spondylitis. A proposal for modification of the New York criteria. Arthritis Rheum..

[19] Garrett S., Jenkinson T., Kennedy L.G., Whitelock H., Gaisford P., Calin A. (1994). A new approach to defining disease status in anky-losing spondylitis: The Bath Ankylosing Spondylitis Disease Activity Index. J. Rheumatol..

[20] van der Heijde D., Lie E., Kvien T.K., Sieper J., Van den Bosch F., Listing J., Braun J., Landewe R., ASAS (2009). ASDAS, a highly discriminatory ASAS-endorsed disease activity score in patients with ankylosing spondylitis. Ann. Rheum. Dis..

[21] Calin A., Garrett S., Whitelock H., Kennedy L.G., O’Hea J., Mallorie P., Jenkinson T. (1994). A new approach to defining functional ability in ankylosing spondylitis: The development of the Bath Ankylosing Spondylitis Functional Index. J. Rheumatol..

[22] Wanders A.J., Landewé R.B., Spoorenberg A., Dougados M., van der Linden S., Mielants H., van der Tempel H., van der Heijde D.M.F.M. (2004). What is the most appropriate radiologic scoring method for ankylosing spondylitis? A comparison of the available methods based on the Outcome Measures in Rheumatology Clinical Trials filter. Arthritis Rheum..

[23] Choi J.H., Kim T.J., Shin K., Choi C.B., Kim J.H., Kim S.H., Kim N.I., Ahn M.J., Jung H.J., Lee K.E. (2014). The reliability and validity of a Korean translation of the ASAS Health Index and Environmental Factors in Korean patients with axial spondyloarthritis. J. Korean Med. Sci..

[24] Kiltz U., van der Heijde D., Boonen A., Braun J. (2014). The ASAS Health Index (ASAS HI)—A new tool to assess the health status of patients with spondyloarthritis. Clin. Exp. Rheumatol..

[25] Essers I., Hiligsmann M., Kiltz U., Bansback N., Braun J., van der Heijde D., Boonen A. (2019). Development of one general and six country-specific algorithms to assess societal health utilities based on ASAS HI. RMD Open.

[26] Min H.K., Lee J., Ju J.H., Park S.H., Kwok S.K. (2019). Predictors of Assessment of Spondyloarthritis International Society (ASAS) Health Index in Axial Spondyloarthritis and Comparison of ASAS Health Index between Ankylosing Spondylitis and Nonradiographic Axial Spondyloarthritis: Data from the Catholic Axial Spondyloarthritis COhort (CASCO). J. Clin. Med..

[27] Kiltz U., Wiatr T., Redeker I., Baraliakos X., Fedorov K., Braun J. (2022). Effects of patient and disease characteristics on global functioning in patients with axial spondyloarthritis in routine care. Semin. Arthritis Rheum..

[28] Smith J.A., Colbert R.A. (2014). Review: The Interleukin-23/Interleukin-17 Axis in Spondyloarthritis Pathogenesis: Th17 and Beyond. Arthritis Rheumatol..

[29] Wu B., Nakamura A. (2022). Deep Insight into the Role of MIF in Spondyloarthritis. Curr. Rheumatol. Rep..

[30] Reveille J.D., Ximenes A., Ward M.M., Deodhar A., Clegg D. (2012). Economic Considerations of the Treatment of Ankylosing Spondylitis. Am. J. Med. Sci..

[31] Kiltz U., Baraliakos X., Karakostas P., Igelmann M., Kalthoff L., Klink C., Krause D., Schmitz-Bortz E., Flörecke M., Bollow M. (2012). The degree of spinal inflammation is similar in patients with axial spondyloarthritis who report high or low levels of disease activity: A cohort study. Ann. Rheum. Dis..

[32] Landewé R.B.M., van der Heijde D. (2021). Use of multidimensional composite scores in rheumatology: Parsimony versus subtlety. Ann. Rheum. Dis..

